# Association of mitochondrial DNA haplogroups J and K with low response in exercise training among Finnish military conscripts

**DOI:** 10.1186/s12864-021-07383-x

**Published:** 2021-01-22

**Authors:** Jukka Kiiskilä, Jari Jokelainen, Laura Kytövuori, Ilona Mikkola, Pirjo Härkönen, Sirkka Keinänen-Kiukaanniemi, Kari Majamaa

**Affiliations:** 1 Research Unit of Clinical Neuroscience, Neurology, University of Oulu, P.O. Box 5000, FI-90014 Oulu, Finland; 2 Department of Neurology and Medical Research Center, Oulu University Hospital, Oulu, Finland; 3 Center for Life Course Health Research, University of Oulu, Oulu, Finland; 4 Unit of General Practice, Oulu University Hospital, Oulu, Finland; 5 Rovaniemi Health Center, Rovaniemi, Finland; 6 Center for Life Course Health Research, University of Oulu, Oulu, Finland; 7 Unit of Primary Health Care, Oulu University Hospital, Oulu, Finland; 8 Healthcare and Social Services of Selänne, Pyhäjärvi, Finland

**Keywords:** mtDNA haplogroup, Exercise dose, Trainability, Low-responder, Military conscript, Population-based cohort

## Abstract

**Background:**

We have previously suggested that some of the mutations defining mitochondrial DNA (mtDNA) haplogroups J and K produce an uncoupling effect on oxidative phosphorylation and thus are detrimental for elite endurance performance. Here, the association between haplogroups J and K and physical performance was determined in a population-based cohort of 1036 Finnish military conscripts.

**Results:**

Following a standard-dose training period, excellence in endurance performance was less frequent among subjects with haplogroups J or K than among subjects with non-JK haplogroups (*p* = 0.041), and this finding was more apparent among the best-performing subjects (*p* < 0.001).

**Conclusions:**

These results suggest that mtDNA haplogroups are one of the genetic determinants explaining individual variability in the adaptive response to endurance training, and mtDNA haplogroups J and K are markers of low-responders in exercise training.

**Supplementary Information:**

The online version contains supplementary material available at 10.1186/s12864-021-07383-x.

## Background

More than half of the inter-individual differences in maximal oxygen uptake (VO_2_ max) is determined by a polygenic effect [1, 2]. In addition, at least 97 genes in nuclear or mitochondrial genomes have been identified to affect VO_2_ max trainability [3], and variation in mitochondria-related genes is associated with exercise response phenotypes [4]. Indeed, mtDNA may be one of the key determinants of VO_2_ max taking into account the fact that aerobic capacity has a greater maternal than paternal inheritance with maternal heritability reaching 28% [5, 6].

Most of the polymorphisms in mtDNA are neutral or nearly neutral, but emerging evidence has suggested that mtDNA is evolving under selective constraint [7]. Even the common population variants of mtDNA have functional consequences and are subject to natural selection [8, 9]. Indeed, associations between mtDNA sequence variation and complex diseases or phenotypes has been found [10], and deleterious mutations in mtDNA are a cause of many mitochondrial disorders [11]. Furthermore, growing evidence suggests that mtDNA haplogroups have an influence on physical performance in athletes [12–16], although the association between elite performance and haplogroup has not been consistent across studies. Differences in ethnic background of the athletes and differences in sport disciplines used for participant selection may at least partly explain this inconsistency [17, 18].

We have previously shown that the frequency of mtDNA haplogroup J and haplogroup K is lower in Finnish elite endurance athletes than in sprint athletes [12, 19]. Moreover, haplogroup K has been found to be infrequent among Polish male endurance athletes [20], and the frequency of haplogroup J is higher in Iranian athletes competing in instant power events or team sports than that in endurance sports [21]. Consistent with these findings subjects with haplogroup J have lower VO_2_ max than subjects with non-J haplogroups [5]. These findings suggest that haplogroups J and K are not favorable in situations, where efficient ATP production is required.

Based on these previous findings, we hypothesized that haplogroups J and K show lower response to exercise training than non-JK haplogroups. Therefore, we analyzed mtDNA haplogroups J and K in a population-based cohort of young Finnish men (*n* = 1036) that attended their compulsory military service. Physical performance of the conscripts was examined in the beginning and end of the service by means of the 12-min Cooper running test and muscle fitness test. The dataset consists of individuals with homogeneous ethnic background and is one of the largest used for analysis of association between mtDNA haplogroups and physical performance. We found that in the end of the military service, excellence in endurance performance was less frequent among subjects with haplogroups J or K than among subjects with non-JK haplogroups.

## Results

Mitochondrial DNA haplogroups J and K were determined in a population-based cohort of 1036 military conscripts. Thirty-nine (3.8%) conscripts belonged to haplogroup J and 40 (3.9%) conscripts to haplogroup K, while the non-JK haplogroups constituted 92.3% (*n* = 957) of the conscripts (Table 1). Physical activity before military service did not differ between subjects with haplogroup J or K and those with non-JK haplogroups (0.635, df=2, *p* = 0.73, G-test). Moreover, seven variables related to body composition and physiology were assessed as possible confounding factors in association analysis of mtDNA haplogroups and physical performance. No difference was found between haplogroups J and K and non-JK haplogroups in these variables among the conscripts (*p* > 0.05, Mann-Whitney U test, Table 2) or among the 237 subjects belonging to the best performing quartile in Cooper test 2 (*p* > 0.05, Mann-Whitney U test, Additional file 1: Table S1). The median MFI did not differ between haplogroups J and K and non-JK haplogroups (*p* > 0.05, Mann-Whitney U test, Table 3).

**Table 1:** Frequency of mtDNA haplogroups in Finnish military conscripts (*n* = 1036)

Haplogroup	N	%
H	475	45.8
HV	4	0.4
V	71	6.8
U	289	27.9
K	40	3.9
J	39	3.8
JT	1	0.1
T	37	3.6
W	57	5.5
I	7	0.7
X	6	0.6
Others	10	1.0

Others= haplogroup Z or other non-European haplogroups

**Table 2:** Clinical characteristics of the Finnish military conscripts harboring haplogroups J and K (*n* = 79) and non-JK haplogroups (*n* = 957)

Variable	Beginning			End		
	Haplogroups J and K	Non-JK haplogroups	***p***-value*	Haplogroups J and K	Non-JK haplogroups	***p***-value*
Body mass index (kg/m^2^)	23.2 (21.3–25.3)	23.0 (21.1–25.8)	0.89	22.9 (21.7–25.2)	22.9 (21.3–25.0)	0.65
Body fat (%)	15.0 (11.8–19.0)	15.0 (10.4–21.4)	0.91	14.2 (11.4–19.0)	14.6 (11.4–18.2)	0.91
Visceral fat area (cm^2^)	54.8 (34.4–74.5)	57.7 (27.1–90.3)	0.83	33.4 (18.8–51.1)	28.7 (9.8–51.3)	0.25
Fat-free body mass (kg)	61.0 (55.8–66.9)	61.3 (56.7–66.6)	0.98	62.4 (56.5–69.0)	61.9 (57.5–67.0)	0.79
Systolic blood pressure (mmHg)	126.3 (119.1–139.5)	127.0 (119.0–138.0)	0.80	126.5 (117.5–136.0)	126.0 (118.0–135.0)	0.53
Fasting plasma glucose (mmol/l)	5.2 (4.9–5.4)	5.2 (4.9–5.5)	0.18	5.3 (5.0–5.5)	5.3 (5.0–5.7)	0.36
Total plasma cholesterol (mmol/l)	3.7 (3.2–4.5)	3.8 (3.3–4.4)	0.43	4.2 (3.6–4.7)	4.2 (3.7–4.8)	0.48

The data was collected in the beginning and in the end of the military service. The values are medians (*interquartile ranges)*. ^*^Mann-Whitney U test

**Table 3:** Results of the Cooper 12-min running test and the total muscle fitness index in Finnish military conscripts

	Haplogroups	Non-JK	***p***-value*
	J and K	haplogroups	
**(A)**			
N	79	957	
Cooper test 1 (m)	2500 (2273–2773)	2500 (2250–2770)	0.89
Cooper test 2 (m)	2700 (2450–2848)	2680 (2470–2900)	0.78
MFI 1 (points)	9.5 (6.0–12.0)	8.0 (5.0–11.0)	0.14
MFI 2 (points)	10.0 (8.0–13.0)	10.0 (7.0–13.0)	0.46
**(B)**			
N	19	218	
Cooper test 1 (m)	3000 (2860–3000)	3000 (2850–3035)	0.81
Cooper test 2 (m)	2960 (2900–3000)	3000 (3000–3070)	0.00019
MFI 1 (points)	13.5 (13.0–15.0)	13.0 (12.0–14.0)	0.06
MFI 2 (points)	14.0 (14.0–15.0)	14.0 (14.0–15.0)	0.63

The data are shown for (A) all conscripts and (B) the best-performing conscripts (1) in the beginning of the service and (2) in the end of the service. The values are medians (*interquartile ranges).* *MFI* total muscle fitness index; *Mann-Whitney U test

The conscripts (*n* = 1036) ran the 12-min Cooper test in the beginning and in the end of the military service, and on both occasions the mean distance covered by subjects with haplogroup J or K did not differ from that covered by subjects with non-JK haplogroups (Table 3). However, there was a difference in the frequency of subjects who covered at least 3000 m in Cooper test 2. Indeed, only 10.5% of the conscripts with haplogroup J or K ran at least 3000 m, while the frequency was 19.5% among conscripts with non-JK haplogroups (4.194, df=1, *p* = 0.041, G-test). In Cooper test 1 there was no such frequency difference between these groups (0.003, df=1, *p* = 0.954, G-test).

**Fig. 1:**
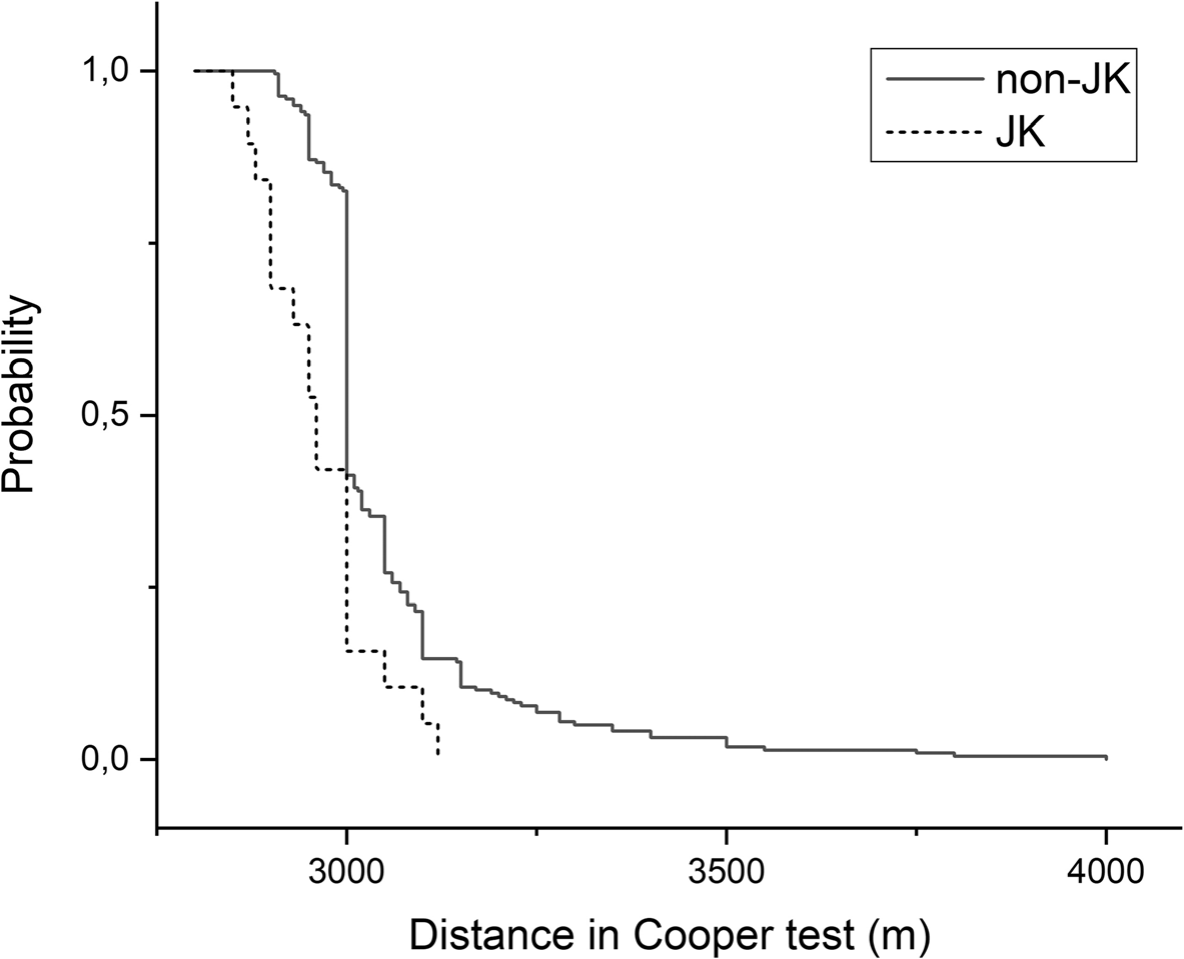
Probability of best-performing conscripts reaching a given distance in 12-min Cooper test in the end of the military service. The data are shown for subjects harboring haplogroups J or K and subjects with non-JK haplogroups

The best performing quartile of conscripts (*n* = 19) harboring haplogroup J or K differed from those with non-JK haplogroups in Cooper test 2 (*p* = 5.8 × 10^− 5^, log rank test, Fig. 1). The median distance covered by those harboring haplogroup J or K was 2960 m in Cooper test 2, while the best performing quartile harboring non-JK haplogroups (*n* = 218) covered 3000 m (*p* < 0.001, Mann-Whitney U test, Table 3). The effect of haplogroups on Cooper distance was shown also in a mixed-model GLM analysis that takes repeated measurements into account (F=4.124, *p* = 0.043, Additional file 2: Table S2), while visceral fat area turned out to be a significant confounding variable (F=17.432; *p* = 3.7 × 10^− 5^). However, visceral fat area affected Cooper distance only in the beginning of the military service (univariate GLM analysis, F=23.813; *p* = 2.0 × 10^− 6^, Additional file 3: Table S3) but not in the end of the service (F=0.517; *p* = 0.473, Additional file 4: Table S4). The main effect of haplogroups J and K and non-JK haplogroups on Cooper test 2 was significant (F=6.298; *p* = 0.013).

No association was found, when conscripts harboring haplogroup J or T were compared with those harboring non-JT haplogroups and when conscripts harboring haplogroup K or U were compared with those harboring non-KU haplogroups (*p* > 0.05, G-test).

## Discussion

We examined a population-based group of healthy young men, who entered their military service. The dose of physical training in the military service is rather standardized and, interestingly, we found an association between mtDNA haplogroups J and K and endurance performance in the end, but not in the beginning, of the military service. The results suggest that subjects with mtDNA haplogroup J or K exhibit lower response to aerobic training intervention. We have previously found that elite endurance athletes harbor mtDNA haplogroups J and K less frequently than elite sprint athletes suggesting that these haplogroups are not favorable in situations, where maximal aerobic performance is required [12, 19]. Our current findings suggest that this association could be due to a low response to training among subjects harboring mtDNA haplogroup J or K.

Responsiveness to exercise training is a continuum and there is a wide inter-individual variation in the response to similar training program. High-responders exhibit exceptionally good response to training, while at the other end of the spectrum, low-responders adapt poorly to training [22]. Previous studies have suggested that some 15% of subjects are low-responders and 15% are high-responders [23]. In accordance, we found that 18.8% of the conscripts covered 3000 m in Cooper test 2. The 3000-m run corresponds to VO_2_ max of 55.78 ml/kg/min [24], which represents superior cardiovascular fitness for the age group that attends military service [25]. Interestingly, we found that only 10.5% of the conscripts with haplogroup J or K reached 3000 m in Cooper test 2, whereas 19.5% of those with non-JK haplogroups covered this distance. Furthermore, the median distance covered by the best performing quartile of the conscripts with haplogroup J or haplogroup K was 40 m less in Cooper test 2 than that of the best quartile with non-JK haplogroups. Statistical analysis showed that none of the clinical and physiological variables had confounding effects on the results of Cooper test 2. Altogether, our data suggested an association of haplogroups J and K with decreased endurance performance among individuals, who train and who pursue maximal performance and, thus, bear similarity to elite athletes.

Physical training is an integral part of the military service and the exercise dose is relatively standardized [26]. The Cooper test results improve during the service [27], but they also reveal a wide variation in the response to training. Genetic factors account for 30–60% of the inter-individual variation in VO_2_ max trainability and several single nucleotide polymorphisms are associated with the low-responder phenotype or high-responder phenotype [3, 28, 29]. Our finding on the association of haplogroups J and K with lower endurance performance following a standard-dose training suggests that these haplogroups are markers of low-responders. Our finding is also supported by a recent study showing that none of 15 high-responders defined by VO_2_ max belonged to haplogroup J [30]. Previously, a study on 20,239 healthy subjects has indicated that age, gender, BMI and physical activity affect cardiorespiratory fitness and explain 56% of the variance [31]. Here, none of these variables differed between military conscripts with haplogroup J or haplogroup K and those with non-JK haplogroups. All of the subjects were men and belonged to the same age group and there was no difference in BMI and physical activity between the haplogroups.

Aerobic training upregulates OXPHOS complexes in the skeletal muscle and [32], furthermore, haplogroup-defining variants can modulate the expression of mitochondrial genomes. Functional studies have shown that cell cybrids harboring haplogroup J contain less mtDNA and synthesize a smaller amount of mtDNA-encoded polypeptides and, hence, display lower oxygen consumption, mitochondrial inner membrane potential and total ATP levels than cybrids harboring haplogroup H [33]. Moreover, cell cybrids harboring haplogroup J1 or haplogroup K1 have been shown to be more sensitive to rotenone, an inhibitor of OXPHOS complex I, than cells harboring haplogroup H1 [34]. Finally, DNA methylation and transcription differ between samples from subjects with haplogroup J and those from subjects with haplogroup H [35]. These results from functional studies provide explanation to our previous finding that haplogroups J and K are rare among elite athletes and our current finding that haplogroups J and K are rare among those that respond well to endurance training.

Conscripts with haplogroup J or K did not differ from those with non-JK haplogroups in the MFI score. Lack of association may be due to the diverse components of MFI score that is composed of measures of endurance performance as well as measures of explosive force production [36, 37]. Indeed, genetic association studies often fail to demonstrate associations between athletic performance and genotype, if the performance phenotype is defined by anaerobic and aerobic tests or power and endurance tests [38, 39].

This is the first study to address the effect of mtDNA haplogroups on training response in a large and rather unselected, ethnically homogeneous population of healthy young men during military service. However, a small proportion of the recruits in Sodankylä Jaeger Brigade may be ethnically Saami. The Saami are considered genetic outliers among European populations. The Saami gene pool is predominantly European with an east Asian contribution of 6% in autosomal genes [40] and 4% in mtDNA [41]. Saami mtDNA pool is characterized by predominance of European haplogroups V and U5b1b1, while a minor proportion consists of eastern Eurasian mtDNA lineages Z1 and D5 [42, 43]. The true number of conscripts with Saami ancestry is not available, as ethnicity is not recorded in Finland. However, the number of Saami speakers is available (Statistics Finland, www.stat.fi) and their proportion among men aged 18–20 years in the catchment population of Sodankylä Jaeger Brigade was 0.3% in 2005. In consequence, the great majority of the conscripts in the cohort was ethnically Finns, and we do not consider that mtDNA from other ethnic groups was a significant confounding factor.

One of the strengths of this study is that the living conditions of the study subjects were rather standardized. The conscripts were housed in the garrison and the service period was structured, so that inter-individual variation in factors such as daily physical activity or caloric intake was relatively small. Training intensity may slightly differ between military branches, but the total time spent on physical training across the branches is approximately 450 h during six months of service [44]. Furthermore, caloric content of the daily meals is rather constant being 3200–3600 kcal/day [45]. The limitations of the study include the fact that approximately 10% of the age group are exempted from military service because of medical reasons [37]. Therefore, our findings may not be generalizable to individuals with pre-existing health-conditions. In addition, as the number of women who enter military service in Finland is low, women were not included in this study and, hence, the results should not be extended to females.

## Conclusions

We have previously found that the frequency of mtDNA haplogroups J and K is lower among elite endurance athletes than sprint athletes suggesting that these genomes are not beneficial in situations, where efficient ATP production is required [12, 19]. Here we showed that this association is detectable also in the general population and, furthermore, that mtDNA variation contributes to the response to endurance training. The best-performing quartile of subjects with haplogroup J or K performed less efficiently than those with non-JK haplogroups suggesting that mtDNA haplogroups are one of the genetic factors that explain variation in inter-individual responses to exercise and that haplogroups J and K are markers of low-responders.

## Methods

### Subjects

Military service is compulsory in Finland for all men over 18 years of age and most men enter the service at the age of 19–20 years. On average, physical training accounts for 40% of the 320 h allotted to the service during the basic training period that consists of activities such as combat skills, marching and sport-related physical training. The dose of exercise is relatively standardized as the training follows a scheduled program and proceeds progressively enabling conscripts to acquire maximal performance capacity by the end of the military service [26, 46]. The duration of the service is 6, 9 or 12 months depending on the branch. Most of the beneficial changes in aerobic performance occurs during the first 6 months of service [27]. Moreover, the greatest improvement in VO_2_ max and muscle strength seem to take place already during the basic training period without further improvement during the later stages of service [47]. Therefore, it is reasonable to consider that all conscripts, regardless of their service duration, reach their peak performance capacity by the end of the service.

Approximately 80% of the male population complete the service, while close to 10% of the age group are exempted due to medical reasons and an additional 8% of the age group attend non-military service [37]. The 1467 conscripts attending military service in Sodankylä Jaeger Brigade in 2005 were invited to the present study and 1160 (79.0%) of them consented, of whom 140 conscripts discontinued the service. The cohort is representative of a rather unselected sample of healthy young men of the age group.

### Clinical and physiological data collecting and assessment of physical performance

Physical activity before military service was assessed by a questionnaire developed by the National Aeronautics and Space Administration’s Johnson Space Center [48]. Each subject was instructed to rate their physical activity on a 0–7 scale during the previous month. The responses 0 and 1 indicated no regular physical activity, 2–3 indicated moderate-intensity physical activity and 4–7 were representative of vigorous-intensity activity [49]. Endurance performance was assessed by the Cooper 12-min running test in the beginning (Cooper test 1) and in the end (Cooper test 2) of the military service [24]. The conscripts were asked to run 12 min with maximal effort. The test was supervised by military personnel and the distance was measured with an accuracy of ±10 m. The subjects who covered at least 3000 m were considered having excellent aerobic fitness [25].

Muscle fitness was assessed by push-ups, pull-ups, sit-ups, trunk extensions and a standing long jump. Each test was scored on a 0–3 scale and muscle fitness index (MFI) was calculated as the sum of the scores. MFI score of 0–4 represented poor, score of 5–8 satisfactory, score of 9–12 good and score of 13–15 excellent total muscle fitness. The assessment was carried out in the beginning of the service (MFI 1) and in the end of the service (MFI 2). The Cooper test and muscle fitness test were completed at least once by 1036 conscripts and on both occasions by 946 conscripts (81.5%).

Clinical and physiological data of the subjects have been described elsewhere [50]. Seven potential confounding variables were measured in the beginning and in the end of the military service for 897 subjects (77.3%) including body mass index (BMI), body fat percentage, visceral fat area, fat-free body mass, systolic blood pressure, fasting plasma glucose level and total plasma cholesterol level.

### Molecular methods

Total DNA was extracted from whole blood using the ABI Prism™ 6100 Nucleic Acid PrepStation with BloodPrep™ Chemistry Kit according to the manufacture’s protocols (Applied Biosystems, Foster City, USA). Restriction fragment analysis was used to detect mtDNA haplogroups J and K (Additional file 5: Table S5) [51, 52].

### Statistical analysis

Statistical analysis was performed with IBM® SPSS® Statistics Version 22 software. Physical activity level before military service was compared between conscripts belonging to haplogroups J and K and non-JK haplogroups with likelihood ratio chi-squared test (G-test). Continuous variables were not normally distributed (Shapiro-Wilk, *p* > 0.05), and therefore non-parametric Mann-Whitney U test was used for statistical comparisons between subjects with haplogroup J or K and those with non-JK haplogroups. The results are shown as medians and interquartile ranges.

The frequency of subjects, who covered at least 3000 m in the Cooper test, was compared between the groups using likelihood ratio chi-squared test (G-test). Furthermore, the Cooper test results and MFI scores were divided into quartiles with lower rank being used for tied values. The results of subjects in the top quartiles were compared between haplogroups J and K and non-JK haplogroups. Kaplan-Meier plots were constructed to visualize the probability of subjects reaching a given distance in the 12-min Cooper test [53, 54], and log rank test was used to estimate the difference between the plots.

To allow the use of parametric tests, logarithmic transformation was applied for non-normally distributed variables, since it produced the closest approximation of normality. The effect of mtDNA haplogroups J and K and non-JK haplogroups on the logarithm of Cooper test results was assessed using univariate general linear model (GLM) ANOVA. In order to control for possible confounding effects of clinical and physiological variables on the Cooper test result, seven clinical and physiological variables (body mass index and logarithms of body fat percentage, visceral fat area, fat-free body mass, systolic blood pressure, fasting plasma glucose and total plasma cholesterol) were included as covariates. In addition, a mixed-model GLM was employed to take into account repeated within subject measurements of the data.

## Supplementary Information

**Additional file 1: Table S1.** Clinical characteristics of the military conscripts belonging to the best quartile in the Cooper test 2.

**Additional file 2: Table S2.** Association of clinical variables and mtDNA haplogroups J and K with Cooper test distance in the best performing quartile of conscripts (Mixed-model repeated measures).

**Additional file 3: Table S3.** Association of clinical variables and mtDNA haplogroups J and K with Cooper test 1 distance in the best performing quartile of conscripts (univariate GLM).

**Additional file 4: Table S4.** Association of clinical variables and mtDNA haplogroups J and K with Cooper test 2 distance in the best performing quartile of conscripts (univariate GLM).

**Additional file 5: Table S5.** Description of the molecular identification of mtDNA haplogroups in Finnish military conscripts.

## Data Availability

The data that supports the findings of this study is available within this paper and its Additional files 1-5. Additional datasets generated during the current study are available from the corresponding author on reasonable request.
