# Supplementary material for: Association of mitochondrial DNA haplogroups J and K with low response in exercise training among Finnish military conscripts
Source: BMC Genomics. 2021 Jan 22;22:75. doi: 10.1186/s12864-021-07383-x (PMC7821635; doi:10.1186/s12864-021-07383-x)
Supplement: Supplementary file 1 — Additional file 1: Table S1. Clinical characteristics of the military conscripts belonging to the best quartile in the Cooper test 2. [file 12864_2021_7383_MOESM1_ESM.pdf]

Table S1. Clinical characteristics of the military conscripts belonging to the best quartile in the Cooper test 2.

**Variable**	**Haplogroups J and K (n=19)**	**Non-JK haplogroups (n=218)**	***p-value**
Body mass index (kg/m^2)	21.8 (20.0–23.9)	22.2 (21.0–23.7)	0.28
Body fat (%)	11.3 (10.3–14.8)	11.9 (9.8–15.3)	0.92
Visceral fat area (cm^2)	20.2 (5.5–32.9)	15.4 (5.0–31.4)	0.48
Fat-free body mass (kg)	60.4 (54.8–66.2)	62.1 (57.5–66.0)	0.46
Systolic blood pressure (mmHg)	129.3 (117.4–142.3)	126.5 (119.0–136.5)	0.55
Fasting plasma glucose (mmol/l)	5.25 (4.88–5.43)	5.30 (4.90-5.60)	0.46
Total plasma cholesterol (mmol/l)	3.95 (3.58–4.40)	4.30 (3.80-4.80)	0.15

The values are medians (interquartile ranges); *Mann-Whitney U test.
