# Supplementary material for: Association of mitochondrial DNA haplogroups J and K with low response in exercise training among Finnish military conscripts
Source: BMC Genomics. 2021 Jan 22;22:75. doi: 10.1186/s12864-021-07383-x (PMC7821635; doi:10.1186/s12864-021-07383-x)
Supplement: Supplementary file 2 — Additional file 2: Table S2. Association of clinical variables and mtDNA haplogroups J and K with Cooper test distance in the best performing quartile of conscripts (Mixed-model repeated measures). [file 12864_2021_7383_MOESM2_ESM.pdf]

Table S2. Association of clinical variables and mtDNA haplogroups J and K with Cooper test distance in the best performing quartile of conscripts.

Source	Numerator df	Denominator df	F	p-value*
Intercept	1	402.944	3348.65	2.53×10^{-197}
Haplogroups JK vs non-JK	1	420.193	4.124	0.043
Body mass index (kg/m^2)	1	421.127	0.4	0.53
Log body fat (%)	1	366.086	0.003	0.96
Log visceral fat area (cm^2)	1	357.205	17.432	3.7×10^{-5}
Log fat-free body mass (kg)	1	420.773	3.455	0.064
Log systolic blood pressure (mmHg)	1	421.893	3.628	0.057
Log fasting plasma glucose (mmol/l)	1	421.867	1.225	0.27
Log total plasma cholesterol (mmol/l)	1	421.731	0.19	0.66

*Mixed-model repeated measures; dependent variable, Logarithm of the Cooper test results.
