# Supplementary material for: Association of mitochondrial DNA haplogroups J and K with low response in exercise training among Finnish military conscripts
Source: BMC Genomics. 2021 Jan 22;22:75. doi: 10.1186/s12864-021-07383-x (PMC7821635; doi:10.1186/s12864-021-07383-x)
Supplement: Supplementary file 3 — Additional file 3: Table S3. Association of clinical variables and mtDNA haplogroups J and K with Cooper test 1 distance in the best performing quartile of conscripts (univariate GLM). [file 12864_2021_7383_MOESM3_ESM.pdf]

Table S3. Association of clinical variables and mtDNA haplogroups J and K with Cooper test 1 distance in the best performing quartile of conscripts.

Source	Type III Sum of Squares	df	Mean Square	F	p-value*
Corrected Model	.013[a]	8	0.002	4.100	$1.7x10^{-4}$
Intercept	0.657	1	0.657	1666.212	$2.1x10^{-90}$
Haplogroups JK vs non-JK	3.7x10-6	1	$3.7x10^{-6}$	0.009	0.92
Body mass index (kg/m^2)	3.4x10-5	1	$3.4x10^{-5}$	0.086	0.77
Log body fat (%)	$2.8x10^{-4}$	1	$2.8x10^{-4}$	0.702	0.40
Log visceral fat area (cm^2)	.009	1	0.009	23.813	$2.0x10^{-6}$
Log fat-free body mass (kg)	.001	1	0.001	2.282	0.13
Log systolic blood pressure (mmHg)	.001	1	0.001	1.511	0.22
Log fasting plasma glucose (mmol/l)	$7.5x10^{-6}$	1	$7.5x10^{-6}$	0.019	0.89
Log total plasma cholesterol (mmol/l)	$1.4x10^{-4}$	1	$1.4x10^{-4}$	0.360	0.55
Error	.068	172	$3.9x10^{-4}$		
Total	2194.884	181			
Corrected Total	.081	180			

*Univariate GLM analysis; [a]R Squared = .160 (Adjusted R Squared =.121); dependent variable, Logarithm of the Cooper test results.
