# Supplementary material for: Association of mitochondrial DNA haplogroups J and K with low response in exercise training among Finnish military conscripts
Source: BMC Genomics. 2021 Jan 22;22:75. doi: 10.1186/s12864-021-07383-x (PMC7821635; doi:10.1186/s12864-021-07383-x)
Supplement: Supplementary file 4 — Additional file 4: Table S4. Association of clinical variables and mtDNA haplogroups J and K with Cooper test 2 distance in the best performing quartile of conscripts (univariate GLM). [file 12864_2021_7383_MOESM4_ESM.pdf]

Table S4. Association of clinical variables and mtDNA haplogroups J and K with Cooper test 2 distance in the best performing quartile of conscripts.

Source	Type III Sum of Squares	df	Mean Square	F	p-value*
Corrected Model	.012[a]	8	0.001	3.952	2.1×10^{-4}
Intercept	0.553	1	0.553	1513.658	7.1×10^{-106}
Haplogroups JK vs non-JK	0.002	1	0.002	6.298	0.013
Body mass index (kg/m^2)	6.9×10^{-5}	1	6.9×10^{-5}	0.19	0.66
Log body fat (%)	0.001	1	0.001	1.631	0.20
Log visceral fat area (cm^2)	1.9×10^{-4}	1	1.9×10^{-4}	0.517	0.47
Log fat-free body mass (kg)	2.5×10^{-4}	1	2.5×10^{-4}	0.693	0.41
Log systolic blood pressure (mmHg)	0.001	1	0.001	2.208	0.14
Log fasting plasma glucose (mmol/l)	0.001	1	0.001	1.525	0.22
Log total plasma cholesterol (mmol/l)	0.001	1	0.001	2.866	0.092
Error	0.088	241	3.7×10^{-4}		
Total	3027.775	250			
Corrected Total	0.1	249			

*Univariate GLM analysis; [a]R Squared = .116 (Adjusted R Squared = .087); dependent variable, Logarithm of the Cooper test results.
